# Influences of Climate Change and Variability on Estuarine Ecosystems: An Impact Study in Selected European, South American and Asian Countries

**DOI:** 10.3390/ijerph19010585

**Published:** 2022-01-05

**Authors:** Walter Leal Filho, Gustavo J. Nagy, Filipe Martinho, Mustafa Saroar, Mónica Gómez Erache, Ana Lígia Primo, Miguel A. Pardal, Chunlan Li

**Affiliations:** 1 Research and Transfer Centre “Sustainable Development and Climate Change Management’’, Hamburg University of Applied Sciences, Ulmenliet 20, 21033 Hamburg, Germany; w.leal@mmu.ac.uk; 2 Department of Natural Sciences, Manchester Metropolitan University, Chester Street, Manchester M1 5GD, UK; 3 Facultad de Ciencias, Universidad de la República (UdelaR), Instituto de Ecología y Ciencias Ambientales (IECA), Oceanografía y Ecología Marina, Iguá 4225, Montevideo 11400, Uruguay; gnagy@fcien.edu.uy (G.J.N.); mgomezerache@gmail.com (M.G.E.); 4 Centre for Functional Ecology-Science for People & the Planet, Department of Life Sciences, University of Coimbra, Calçada Martim de Freitas, 3000-456 Coimbra, Portugal; fmdm@ci.uc.pt (F.M.); ana.primo@uc.pt (A.L.P.); mpardal@uc.pt (M.A.P.); 5 Department of Urban and Regional Planning, Khulna University of Engineering & Technology (KUET), Khulna 9203, Bangladesh; saroar.mustafa@yahoo.com; 6 Directorate of the Environment (DINAMA), Climate Change Division (DCC), NAPA Coastal Areas 25 de Mayo, Montevideo 11000, Uruguay; 7 Center for Geopolitical and Strategic Studies & Institute for Global Innovation and Development, East China Normal University, Shanghai 200062, China; 8 State Key Laboratory of Cryospheric Science, Northwest Institute of Eco-Environment and Resources, Chinese Academy Sciences, Lanzhou 730000, China; 9 School of Urban and Regional Sciences, East China Normal University, Shanghai 200241, China

**Keywords:** climate threats, species distribution, adaptation measures, estuaries

## Abstract

It is well-known that climate change significantly impacts ecosystems (at the macro-level) and individual species (at the micro-level). Among the former, estuaries are the most vulnerable and affected ecosystems. However, despite the strong relations between climate change and estuaries, there is a gap in the literature regarding international studies across different regions investigating the impacts of climate change and variability on estuaries in different geographical zones. This paper addresses this need and reviews the impacts of climate change, variability and extreme weather on estuaries. It emphasises the following: (i) a set of climate parameters governing estuarine hydrology and processes; and (ii) a sample of countries in Asia (Bangladesh), Europe (Portugal) and South America (Uruguay). We reviewed the influences of the climatic drivers of the estuarine hydrology, ecological processes and specific species in estuarine communities across the selected geographical regions, along with an analysis of their long-term implications. The key results from the three estuaries are as following: (i) Hilsa fish, of which the catches contribute to 10% of the total earnings of the fishery sector (1% of GDP), are affected by climate-forced hydrological and productivity changes in the Meghna; (ii) extreme droughts and short-term severe precipitation have driven the long-term abundance and spatial distribution of both fish larvae and juveniles/adults in the Mondego; and (iii) the river inflow and fluctuations increases since the early 1970s have contributed to variations in the salinity, the stratification, the oxygen, nutrient and trophic levels and the spatial pattern for the life stages of planktonic species, fish biomass and captures in the Rio de la Plata. The results suggested that immediate action is needed to reduce the vulnerability of estuaries to climate stressors, mainly the changing river flows, storms and sea-level rise. As a contribution to addressing current problems, we described a set of adaptation strategies to foster climate resilience and adaptive capacity (e.g., early-warning systems, dam management to prevent overflows and adaptive fisheries management). The implications of this paper are two-fold. Firstly, it showcases a variety of problems that estuaries face from changing climate conditions. Secondly, the paper outlines the need for suitable adaptive management strategies to safeguard the integrity of such vital ecosystems.

## 1. Introduction: An Overview of Climate Change Trends and Their Impacts on Estuaries

Recent studies have revealed that increases in the average global temperature, erratic precipitation regimes and frequent and high-magnitude weather extremes [1,2] threaten the natural environment [3,4,5]. For example, the globally averaged combined land and ocean surface temperature data show a warming of 0.99 °C (0.88 °C over the ocean) between 1850–1900 and 2001–2020 [2]. Averaged over the mid-latitude land areas of the Northern Hemisphere, precipitation has increased (high confidence after 1951); for other latitudes, area-averaged long-term positive or negative trends have low confidence [6].

The global mean sea level (GMSL) very likely rose at a mean rate of 1.7 mm yr^−1^ between 1900 and 2010 and at a rate of 3.2 mm yr^−1^ from 1993 to 2010 [7]. During the period 1901–2018, the GMSL increased by 0.20 m. The average rate of the sea-level rise (SLR) was 1.3 mm yr^−1^ between 1901 and 1971, increasing to 1.9 mm yr^−1^ between 1971 and 2006 and further increasing to 3.7 mm yr^−1^ between 2006 and 2018 (high confidence). Human influence has been very likely the primary driver of these increases since at least 1971 [2].

The global surface temperature change at the end of the 21st century is likely to exceed 1.5 °C for all Representative Concentration Pathways (RCPs) scenarios, except for RCP2.6 (e.g., 2 °C for RCP6.0 and RCP8.5 [6]). Furthermore, the IPCC AR-6 WG 1 Report states that by 2081–2100 the average will very likely exceed 2 °C and 3 °C in the intermediate (SSP2-4.5) and very high (SSP5-8.5) emission scenarios when compared to the 1850–1900 average [2]. Besides, all scenarios project a very likely increase of 1.5 °C or more for the near-term period (2021–2040).

Regarding the regional changes in hot extremes, heavy precipitation, and ecological droughts, the IPCC [2] produced sub-regional hexagons, e.g., South-Eastern South America (SES), Western and Central Europe (WCE) and the South Asia (SAS), corresponding to the Rio de la Plata (RdlP), Mondego and Megnha estuaries, respectively. The hot extremes and heavy precipitation increased in the three sub-regions; only WCE showed an agreed decrease in droughts.

Climate change impacts marine ecosystems worldwide [2,7,8,9]. Indeed, estuaries are among ecosystems most threatened by climate change, with low-lying coastal areas and small islands, mainly by the SLR, storms and changes in rainfall [1,2,7,8]. In this regard, Kelman pointed out how to depoliticise development challenges distracted by climate change for small islands, e.g., emphasising the hazard and shifting focus away from opportunities for reducing vulnerability [10], which are helpful in estuaries under uncontrollable climatic threats in the near term (e.g., 2021–2040).

Changes in critical hydroclimatic processes will affect freshwater (FW) and marine ecosystems [11], where estuaries form a transition zone. Estuaries are semi-enclosed transition zones between rivers and sea, encompassing coastal habitats where saltwater from the ocean typically mixes with FW from rivers or streams [12,13,14]. Seawater intrusion in estuaries is mainly due to tides and buoyancy (related to the density difference between seawater and river water). Thus, it determines the habitats and species that can develop in these environments. Besides, density-driven currents and salinity play a role in estuarine turbidity and sedimentation [15].

Estuaries provide biologically productive nursery habitats for fish while buffering many coastal communities from the impacts of storms and SLR. They are filter zones of sediments and pollutants through biological processes, also offering a basis for millions of people’s livelihoods [16,17,18,19,20]. The structure, function, biodiversity and services of estuarine ecosystems are vulnerable to the impacts of changes in precipitation patterns, sea surface water temperature (SST), acidification, SLR (including erosion, flooding, habitat contraction, loss of functionality and biodiversity and salinisation), the frequency and severity of weather extremes and non-climate stressors (e.g., agriculture, overfishing and eutrophication). The heavy precipitation increased in (at least medium confidence) SES, WCE and SAS regions, where the RdlP, Mondego and Megnha estuaries are located respectively. Only WCE shows an agreed increase regarding the droughts, while there is no agreement for SES and SAS [9]. Although these impacts depend on the estuarine geomorphology and management conditions, the residual and potential impacts would undermine estuaries’ ecological, economic and aesthetic values while heightening management costs [1,8,11,18,20,21,22]. Hence, understanding the effects of climate change and variability on estuaries’ biophysical conditions and sensitivity is vital for an informed and proactive response. Because of the high natural variability and broad, but limited, coping capacity of estuarine ecosystems and the species living/visiting within them, they are valuable indicators of coupled climate–environmental changes [23,24,25,26].

This paper aims to the following objectives: (i) describing the main climatic threats and impacts on river-dominated estuarine environments and species distributions; (ii) presenting three examples of river estuaries—the Meghna River in Bangladesh, the Mondego River in Portugal and the RdlP River in Argentina-Uruguay; and (iii) presenting their adaptation actions.

The article structure is shown as follows. Section 2 describes the materials and methods, explaining the focused narrative literature review approach and the descriptions of the three estuaries. Section 3 presents a summary focused on the first objective, i.e., describing the primary physical determinants of estuarine hydrology in general and how they influence ecological processes. Section 4 presents the review of results for the three studied estuaries. Section 5 presents the discussion, focusing on climate adaptation. Finally, Section 6 is the conclusion.

## 2. Research Methods: Approach and Estuaries Descriptions

### 2.1. Approach

The analytic framework of this review paper explores how the changing and variable climate drivers the following: (i) affecting the estuarine hydrological processes, the environment and resources; (ii) affecting the hydrological processes and resources in the Meghna, Mondego and RdlP River estuaries; and (iii) investigating how managers can cope with these external drivers. The implicit hypothesis is that these drivers are (almost) uncontrollable in the near term (e.g., 2021–2040), and the question addressed guiding this review is “How do rainfall and river flow variability and changes affect the hydrological structure and processes governing the environment and biological resources in the three studied estuaries?”.

We followed a narrative literature-based review approach (including published sources, grey literature (e.g., reports), updates and synthesis that were focused on the three objectives as following: (i) summarising the main climatic threats and impacts on river-dominated estuaries and species distributions; (ii) presenting three examples of river estuaries—the Meghna River, the Mondego River and the RdlP River; (iii) presenting their adaptation actions.

**Figure 1:**
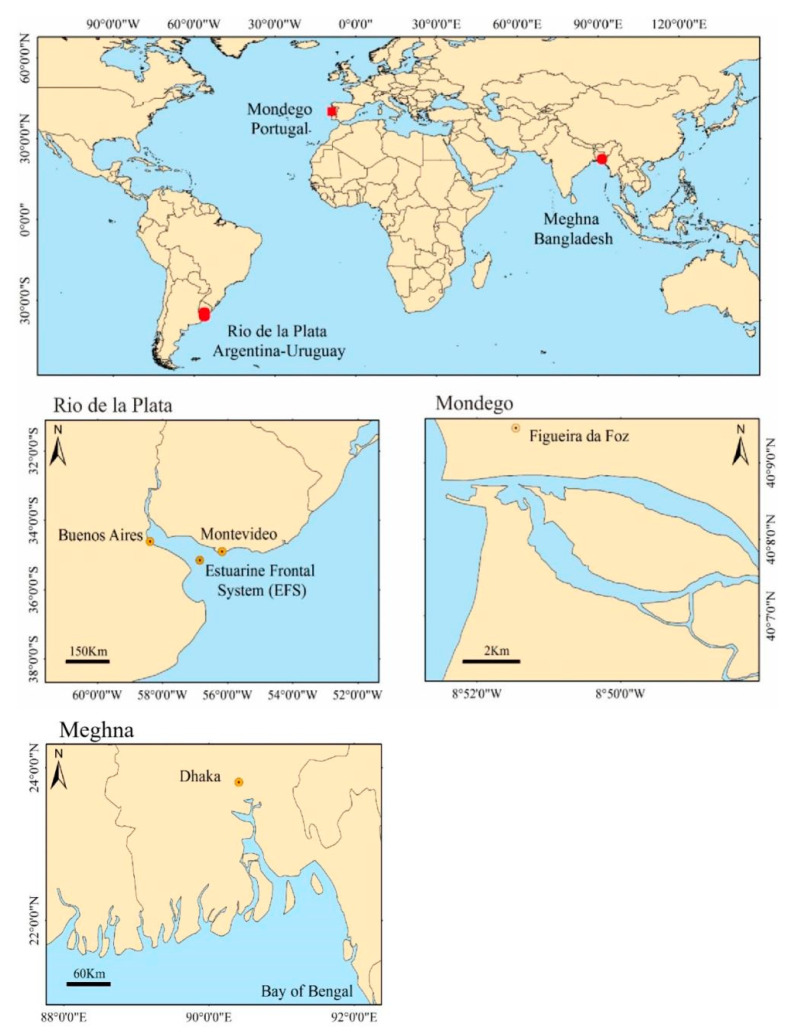
The geographical locations (above) and detailed descriptions of the three estuaries, i.e., Rio de la Plata (RdlP) in Argentina-Uruguay, Mondego in Portugal and Meghna in Bangladesh.

Based on the need for research that may improve the understanding of vulnerability, impacts and adaptation in estuarine ecosystems under current climate variability and future climate change, we presented, in this section, a succinct description of the three studied river estuaries (Figure 1). Section 3 deals with objective 1—describing the main climatic threats and impacts on river-dominated estuarine environments and species distributions, focusing on a literature review of the climate drivers of estuaries’ main hydrological and ecological processes. The results presented examples of climate drivers focused on how river flow variability and extremes affect the hydrology of the three estuaries and their influences on plankton, fishes and fisheries. Finally, in the discussion, we summarised the main adaptation strategies followed in the studied systems.

The rationales for choosing this set of estuaries are the following. Firstly, they originate from three continents, with various climatic impacts and adaptation strategies. Secondly, they illustrate the magnitude of the riverine influence and drivers of climate variability such as the North Atlantic Oscillation (NAO), the El Niño Southern Oscillation (ENSO) and monsoons, as well as expected climate changes. Thirdly, they provide valuable ecosystem services for sustainable development.

Although no broad generalisations are possible, the three studied estuaries offer insights into the nature of various climate threats and impacts on estuaries across the three distinct geographical regions.

### 2.2. Estuaries Description and Research Methods

The descriptions of the Meghna (Ganges delta in Bangladesh), Mondego (Central Portugal) and RdlP (Argentina-Uruguay) River estuaries are summarised in Table 1.

This paper is based on several sources and periods: for the Meghna estuary, the data covered the period 2000–2018 [29,32,41,42,43,44]; for the Mondego estuary, data covered the period of 2003 to 2009 for fish larvae and from 2003 to 2015 for juvenile/adult fish [12,45]. For the RdlP estuary, the historical environmental and hydrological data ranged from 1981 to 1999 (e.g., [37]), and for hydrology, plankton and fisheries from the 2000s to the present [46,47]. In addition, recent climatic, environmental and hydrological data were included (e.g., [38,39,40,48]).

## 3. Literature Review of Climate Threats and Impacts on Estuary Ecosystems

This section reviews some concepts about climate threats and impacts on estuaries in general. Then, we summarised how current estuarine dynamics would be (qualitatively) affected by future increased variability and changes in the river inflow (Q_F_), the wind regime, the SLR, the salinity distribution and stratification, the salt-front location, the environmental changes and the species distribution (examples from plankton and fishes, particularly those migrating between seawater and FW or vice versa to spawn and/or to complete their life cycles).

These parameters were chosen based on data availability and relevance to estuarine ecosystems. Box 1 summarises some of the main estuarine variables and processes, and Table 2 summarises the hydrological processes, their climate forcings, the impacts of current climate variability and near-future changes (2021–2040).

Box 1. **Summary of estuarine variables and processes (modified from Glamore et al. [49]).**

Hydrodynamic (and mixing) processes [49]
Tides cause the regular periodic movement of waters.
Gravitational forces can affect estuarine water circulation due to the gravity acting on the density differences in the estuary [49]. Gravitational circulation driven by river discharge and density gradients dominates in many estuaries, or there is a competition between the tidal forcing and the river discharge [50].
Coriolis forces caused by the rotation of the Earth about its axis may affect large estuaries, because inward and outward currents displace their flow directions to the right/leftward (in the Northern/Southern hemispheres).
Waves affect shores, marginal shoals and shallow estuaries.
Wind forces can significantly modify circulations and turbulent mixing in wider environments. Strong winds may cause storm surges in surface waters that induce large flow volumes. Wind also impacts the levels of stratification and mixing within the estuary. Wind energy influences large, open, and shallow estuaries than deep, narrow ones.
Water quality processes [49]
The temperature influences the rate of plant photosynthesis and aquatic organisms’ metabolic rates.
The salinity controls the type of species that can live in an estuary and influences physical and chemical processes such as flocculation and the amount of dissolved oxygen (DO) in the water column.
The suspended material (SPM) level influences the turbidity in the water column.
DO is the oxygen level available to support estuarine ecology.
Nutrients: In particular, nitrogen and phosphorus are key water quality parameters, as they have significant direct or indirect impacts on plant growth, oxygen concentrations (including hypoxia), and water clarity and sedimentation rates, ecosystem metabolism [50] and eutrophication.
Acidity and alkalinity are essential to ecosystem health, because most aquatic plants and animals are adapted to a specific pH range and alkalinity. Therefore, sharp variations outside of this range can be detrimental.

It is widely accepted that estuaries are among the most important, and yet the most fragile, ecosystems, which experience frequent disturbances, ranging from short-term tidal water levels and salinity changes to longer-term climatic changes due to the ENSO/ the NAO, as well as extreme events such as floods, droughts and storm surges. In addition, the ENSO and the NAO have been related to numerous long-term ecological changes in the marine environment, including several estuarine fish stocks [1,8,52].

Estuarine spatial patterns change over time scales (hours, days, months and years) of variability associated with variations in tides, wind and river inflow and the exchange with coastal waters. In these areas, the spatial distribution of motile organisms is shaped by species-specific adaptations to different salinity ranges and by behavioural responses to environmental variability [53].

A recent study [9] highlights the rapid changes occurring in estuarine environments. The authors found that the temperature and acidification increases in Southeastern Australian estuaries over the last 12 years occurred faster than those predicted by the regional ocean or atmosphere models and more rapidly in small and shallow estuaries with enclosed entrances and longer retention times.

Table 2 summarises the main estuarine hydrological processes and the direct and indirect impacts of climate variability or near-future change (2021–2040) relevant to this article: (i) rainfall and subsequent river inflow; (ii) tides and SLR; (iii) surface heat budget (temperature, evaporation and solar radiation); (iv) wind; and (v) acidification.

## 4. Results

This section presents the main hydrological, environmental features, species distribution and observed and expected environmental changes associated with climate threats of the studied estuaries.

### 4.1. Meghna Estuary

The assessment of Bangladesh’s climate-related vulnerability in terms of the change in fish capture focused on three components of exposure: (1) SLR leading to salinisation; (2) storms and cyclones resulting in an increased height of storm surges; and (3) consecutive wet days causing flash floods and riverine floods [32].

For instance, during the dry period, phytoplankton’s genera decrease to only 16 from >30 in monsoon. This decline in biodiversity and productivity affects the breeding/spawning ground of the anadromous planktivore Hilsa fish (Tenualosa ilisha), of which the catches contribute to 10% of the total earnings of the fishery sector (1% of GDP), because individual plankters are susceptible to changing hydrodynamics [41,42,43,44].

### 4.2. Mondego Estuary

Extreme droughts and short-term severe precipitation have occurred intermittently in Central Portugal over the last 15 years. Furthermore, the high variability in precipitation and runoff (Q_F_) resulted in the displacement of the salinity gradient upstream during droughts (May 2004, September 2008 and December 2011) and downstream during floods, which affected the estuary’s bio-productivity [34,35,64]. These factors have driven the long-term abundance and spatial distribution of fish larvae and juveniles/adults, highlighting the significance of rainfall and Q_F_ as the inputs of organic matter and chemical cues to the adjacent coastal area, which attracts larvae and juveniles towards sheltered estuarine areas [65].

Drought years are characterised by a higher abundance of marine stragglers (MS; see complete classification by estuarine habitat use patterns, i.e., ecological guilds [66]) due to the increased salinity in the lower estuary. In contrast, higher rainfall years were associated with higher abundances of freshwater (FW), catadromous (CA), estuarine residents (ERs), omnivourous (OV), and marine juvenile (MJ) species. The abundances of marine stagglers (MS), MJ, larvae (L) and specific species [67] are negatively correlated with the NAO, mainly through indirect effects on SST, wind and current circulation patterns that are directly linked to most stages of the organisation of fish communities, but at different rates and extents [67].

The significant Pearson correlations (r) between the interannual NAO variability, several environmental descriptors and the ecological guilds were summarised as follows:

1. NAO: MS-L (–0.7); MJ-L (–0.4); MS (–0.5);
2. Precipitation: runoff (0.9), salinity (–0.8); FW, CA-L and ER-L (0.8); OV (0.6); MS (−0.4);
3. Runoff: ER-L (0.8); CA, CA-L and MS (–0.5);
4. Salinity: FW, CA-L and ER-L (–0.8); CA and OV (–0.6); MJ-L (–0.6); ER (–0.4).

### 4.3. RdlP Estuary

The total river inflow has increased since the early 1970s, with significant differences between ENSO phases. The wind stress showes fluctuations in both phases, being less intense during El Niño (EN), during which the increased precipitation and the maximum Q_F_ were observed contributing to variations in salinity, vertical water stratification, decreased clarity, DO_2_, nutrients, and the (outward) location of the estuarine frontal system (EFS). Nevertheless, several La Niña-related droughts have occurred (e.g., 1999–2000, 2004, 2008–2009 and 2018), with increased seawater intrusion and eutrophication symptoms within the inward displaced EFS [37,38,47,48].

The horizontal gradient and vertical stratification of salinity determine the spatial pattern for life stages of planktonic species [46] and fish biomass, as the shallow and FW areas are the preferred habitats of larvae [47]. During the breeding season (spring–summer), adults enter the inner part of RdlP for spawning. Variations in Q_F_ and wind patterns affect the spatial extent of estuarine water, influencing the life-history stages and fish assemblage composition. Seasonal changes in the species composition are related to migration because of salinity and temperature variations and reproductive migrations to spawning and mating areas [47].

## 5. Discussion

### 5.1. Climate-Induced Changes in FW Flow into Estuaries

Medium- to long-term changes in the selected estuaries are mainly driven by climate-induced variability in precipitation and FW flow regimes, which are linked to estuarine productivity, vertical stratification, location of estuarine fronts and the consequent extension of river plumes into coastal areas.

In the Meghna estuary, Bangladesh, drier conditions during winter months will expectedly lead to a low flow regime, a tidal and wave-induced increase in seawater intrusion, an increased SST (<20 °C during dry winter, as compared to the monsoon month’s average of 26 °C), fewer nutrients and a lower planktonic productivity, all of which would affect the current production of Hilsa fish (≈5 million metric tons) and the livelihood of 0.7 million families who are directly/indirectly dependent on the Hilsa value chain (10% of the coastal population). Furthermore, this decrease in fish capture-alongside agriculture and livestock production decreases due to climate-related salinisation in rivers and soils will further affect the poor and vulnerable population in the southern coasts of Bangladesh [32]. Therefore, there is an urgent need to address the Meghna River estuary’s challenges through research and policy attention, including transforming the existing management regimes to an ecosystem-based approach, particularly fishery co-management [44].

In the Mondego, Portugal, while the NAO exerted an indirect control of climatic patterns, monthly variations in rainfall and runoff are the main drivers of fish and larvae abundance in the estuary. ERs and marine-estuarine-dependent (and commercially important) species (e.g., European sea bass, European flounder and common sole) increase the abundance with higher rainfall/runoff due to the increased productivity. Coastal fisheries landings from the latter species also benefit from such conditions [68]. FW species are absent during drought years. In contrast, marine straggler species (including sub-tropical species) benefit from an inward flow of saltwater into the estuary, expanding their available habitats and increasing the total species number within the estuary [34,69].

Further reductions in the intensity and frequency of rainfall will decrease the river runoff into coastal areas and affect the recruitment of commercially important species, of which the juveniles depend on estuaries due to lower planktonic and benthic productivities and the reduction in the extent of river plumes in coastal areas [35,64]. An increase in the sea surface temperature may also impact the early life cycle of cold-temperate species of which the southern distribution limit is the Portuguese coast [69].

In the RdlP estuary (Argentina-Uruguay), environmental variations are likely to modify plankton and fish distributions and abundances. The expected rainfall increase and southeastern winds and sea-level increase for 2050 will have contrasting effects as following: decreased/increased seawater intrusion/salinity and inward/seaward displacement of the EFS [38,39]. Together with the increased water temperature, they would affect the mixing-stratification cycle, primary productivity and oxygen levels in the estuarine front [37,38]. These environmental variations are likely to modify plankton and fish distributions and abundances, thus affecting fisheries’ resources availability and the sustainability of fishing communities [47].

### 5.2. Cross-Comparison and Lessons Learned

Despite the high natural variability of estuaries and the high coping capacity of their habitats and species, extreme floods and droughts induce changes in salinity, spatial and temporal components and the vertical mixing-stratification structure, which, together with the increased CO_2_ concentration (warming and acidification) and the SLR, affect productivity, oxygen levels, species distribution and fisheries sustainability. The three estuaries provide insight into the high variability and the current and expected impacts of the climate variability, change and SLR.

The Meghna is an example of how the increased runoff and windstorms will further impact (particularly under the SLR) their communities’ ecosystem services and livelihood. At the same time, the Mondego and the RdlP also showcase how increased extreme droughts affect the habitat availability and the sustainability of fisheries. Particularly interesting is that this occurs within a long-term increasing trend of rainfall and river inflow in the latter case. In the case of the Mondego estuary, the expected decrease in rainfall (in the WCE region) would increase the occurrence of droughts. Our results point to a differential effect of changing climate scenarios at different time scales, but with a similar impact on fisheries production by mediating fish recruitment success due to changes in primary productivity and trophic linkages, survival of larval stages and the availability and quality of estuarine nurseries [70].

These results suggested some thresholds have been reached; therefore, adaptation strategies based on continuous monitoring and evaluations for flexible and adaptive risk management are needed. The development of the integrated National Adaptation Plans of Action (NAPAs) seems to be an efficient platform for these initiatives [71].

**Figure 2:**
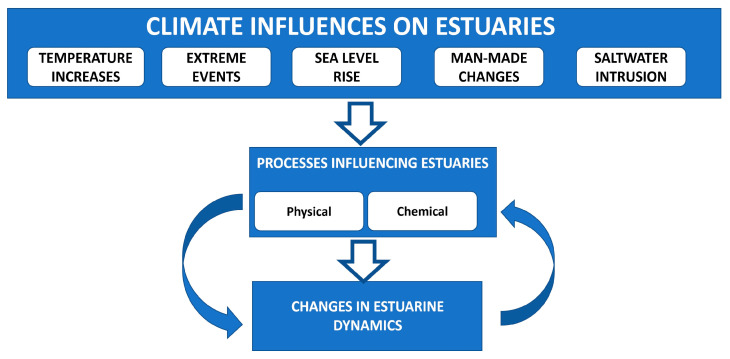
The climate influences and the change in estuarine dynamics and habitat of the three estuaries: the RdlP, Argentina-Uruguay; Mondego, Portugal; and Meghna, Bangladesh.

Figure 2 summarises some of the climate influences on the studied estuaries and the change in estuarine dynamics and habitats.

### 5.3. Building the Climate Resilience of Estuarine Environments through Adaptation Actions

Although the estuarine environment will be influenced by climate change in the future, there is a significant uncertainty about how those changes will exactly unfold. Thus, forecasting cannot be just based on patterns of historical change. Therefore, ecosystem management is critically dependent upon a continuing flow of information from observations to measure, understand and anticipate future changes along the world’s coastlines [56].

An interesting example of an integrated and flexible new adaptation strategy is the Australian decision support framework CoastAdapt (https://coastadapt.com.au; accessed on 25 October 2019), which was developed to understand the impacts of climate change and provide possible response options, based on datasets on historical events, present-day sensitivity, future climate extremes, the SLR and inundation and a risk-management framework on a basis of monitoring and evaluation [72].

Despite the unpredictability of changing environmental conditions, several adaptation measures can be implemented to protect and enhance the ecosystem services provided by estuaries.

In the Meghna estuary, hydrological, sedimentological and fisheries management activities are undertaken. A framework of transboundary cooperation (with India) is in effect to augment the dry season flow. In the wet season, sediment-laden flow is diverted to deeply flooded back swamp areas [73] to help protect the Hilsa fish’s spawning grounds. Other measures such as the building of embankments, maintenance of sluice gates and coastal forestation contribute to reducing disasters, but their roles in species conservation are yet to be studied. Urgent measures are also needed to halt the growing problem of saltwater intrusion and the rapidly increasing population pressure [30,32].

In the Mondego area, the high variability in river runoff was pointed out as one of the main drivers of local variability for estuarine larval and fish communities [34,35]. Hence, improving water management upstream can assist in buffering the effects of low precipitation in a broad time scale, ensuring sufficient FW flow that supports the nursery function of the estuary, combined with the ongoing development of climate-change adaptation plans [36,74].

Implementing adaptation plans and effective measures in the RdlP Basin and estuary is complex due to its dimensions, dams and multi-country transboundary nature [37,39].

However, paying more attention to prevention and readiness by improving observation–early-warning–response systems, complemented by suitable government policies, the following measures may also be considered and implemented, as also suggested by [39,48,72], among others:

(a) Adaptive fisheries management, ecosystem-based approach and fishery co-management to reduce the pressure on fish stocks;
(b) Dam management to prevent overflows;
(c) In estuaries, participatory scenario planning defines adaptation actions under uncontrollable factors and extreme events (e.g., extratropical cyclones and severe droughts);
(d) Adjustments in coastal management plans to cater to the estuaries’ increased fragility (e.g., limiting new construction sites or steering economic activities to minimise their impacts on estuary ecosystems).

These measures are only some examples of some of the actions needed. They need to be combined with other initiatives that consider many physico-chemical processes that characterise estuaries and the physical ones, such as river discharges and sediment dynamics. If implemented, they may help reduce the vulnerability and increase the resilience of estuarine ecosystems to the impacts of a changing climate.

## 6. Conclusions

This paper has presented an analysis of the influences of climate change (warming and SLR), variability (seasonal, the ENSO and the NAO) and extreme weather (storms, droughts floods) on the following: (i) estuarine hydrological and ecological processes; and (ii) a sample of estuaries across three distinct geographical regions. It has illustrated that, even though estuaries provide a range of essential ecosystem services in their respective regions, they are all under various degrees of pressure due to climatic change and variability. A common feature of the three studied systems is that their hydrology, ecology and fish capture is severely affected by river flow changes, especially droughts.

Despite the highly different climatic, socioeconomic and ecological contexts of the studied estuaries, there are a few standard helpful adaptation measures. In addition to improving climate prognosis and early warning systems, water management upstream to reduce the risks of abrupt and severe changes in FW flows and adaptive management of fisheries are crucial strategies for reducing the ecological and economic impacts of a changing erratic and less predictable climate.

Since estuarine habitats support a wide range of species, from microorganisms to crustaceans, fish, shorebirds and marine mammals, combined with their economic, environmental, climatic (CO_2_ sink) and recreational value, there is a need for mechanisms that may help to monitor and evaluate estuarine conditions over more extended periods by using, for instance, numerical models. These may help measure current and estimate future changes in water volumes, salinity or impacts on individual species under projected increases in precipitation and discharge.

Although this paper has a limitation in the sense that only three sites were investigated, it provides a framework upon which a larger sample of estuaries worldwide may be studied. Due to the complexity of estuaries and many ways that climate change, variability and extreme weather may negatively influence them, efforts towards their conservation must consider a combination of technical and management issues and sound policymaking to afford them with an adequate level of protection.

## Figures and Tables

**Table 1:** Hydrological features of the studied estuaries.

Meghna	Mondego	RdlP
Large (1500 km^2^) micro- to macro-tidal (tidal amplitude: <2 m to >4 m) vertically mixed system. During monsoon/post-monsoon (May–July/August–October) and dry period (January–March), the average rainfall is 300 mm/month and 75 mm/month, respectively. Freshwater inflows (Q_F_) reach over 100,000 m^3^/s (40,000–80,000 m^3^/s throughout the monsoon/post-monsoon periods and 3000–10,000 m^3^/s during the dry months [27,28]. Due to its low elevation and high subsidence, the estuary is vulnerable to the monsoon/post-monsoon Q_F_, the water-level increase [29] and the sea-level rise (SLR; >1 mm yr^−1^, +>1 cm in 10 years [30]; 0.32–0.88 m by 2050–2100 [31,32]), and likely increases in water flow due to precipitation extremes [33].	Small (3.4 km^2^) meso-tidal (1–3 m tidal amplitude) and lightly stratified system characterised by dry, warm summers and mild, rainy winters. The Q_F_ was over 180,000 dam^3^, with the average driest years being 67,000 dam^3^ (May 2004, September 2008 and December 2001). A low Q_F_ is observed during warm summer months [34,35,36]. Since the 1930s, interventions to regulate river flow have changed hydrodynamics. The expected SLR by 2050–2100 is 0.30–0.45 m [2].	Huge (38,000 km^2^) micro-tidal (tidal amplitude: <0.5 m) partially stratified system. The total Q_F_ (≈25,000 m^3^/s) varies from <20,000 m^3^ s^−1^ to >30,000 m^3^ s^−1^ during dry/wet years associated with La Niña/El Niño events, respectively [37,38]. The stratification/mixing cycle and the estuarine front (EFS) location are controlled by the wind, Q_F_ and ENSO forcings on daily, seasonal and interannual time scales [37]. The expected SLR by 2050 is 0.3 m [39,40].

**Table 2:** Summary of estuarine hydrological processes, including effects and sensitivity of/to climate variability and change, current and near-future (2021–2040) threats from climate change and variability to estuaries, direct impacts (physical and chemical) and indirect impacts (ecosystem, species and communities). The magnitudes of impact (expert judgment based on the references) were classified as following: weak (↓,↑); moderate (↓↓,↑↑); significant (↓↓↓,↑↑↑); change (∆).

Estuarine Hydrology	Related Climate Forcings	Direct Impacts (Physical and Chemical)	Indirect Impact (Ecosystem, Species and Communities)	References
Q_F_	Rainfall and river discharge (Q_F_) Heavy rains and floods Poor rains and droughts	Horizontal salinity differences affect estuarine circulation and the salt-front location following the increase/decrease of Q_F_, respectively. ↑↑↑downward/upward displacement of the salt front; ↑↑ horizontal salinity gradient; ∆ in physical mixing characteristics: ↑/↓stratification; ↑-↑↑ /↓- ↓↓salinity, saltwater area and volume (A-V) ∆ Turbidity ∆ Nutrients and trophic status ∆ DO_2_ and hypoxia ∆ Primary productivity and higher trophic levels	Salinity controls the species living in an estuary and influences dissolved oxygen (DO_2_), hypoxia, the phytoplankton growth rate and primary productivity (CO_2_ sink). Nutrients (N and P) directly or indirectly impact plant growth, (DO), pH, clarity, primary productivity, eutrophication and ultimately CO_2_ emissions.	[7,34,46,49,50,51,52,53,54,55,56,57,58,59]
Tide	Sea-level rise	Altered hydrodynamics Periodic inward/outward movement of waters Regulate vertical mixing, stratification and water level. ↑ Salinity and saltwater A-V; ↑ Inundation and erosion; ↑saltwater further upstream; ↑tidal volume flow ∆ Circulation and sediment transport.	↑ ∆ Habitat and species distribution Estuarine habitats migrate landwards. ∆ Nutrient dynamics	[49,50,54,60]
Tempe- rature	Global warming	↑ Rate of plant photosynthesis, respiration (R) and the metabolic rates of organisms. ↓ DO_2_; ↓ stratification; ↓ pH	↑ Ecosystem Metabolism (↑R) and ∆ trophic status	[51,58,61]
Winds	Wind increase/ decrease	∆ Circulation, mixing, stratification and salt-front displacement, especially in large, wide and shallow estuaries Strong winds induce large flow volumes and may cause storm surges.	Controling clarity and primary productivity	[49,50,53]
Increased atmospheric CO_2_	↑water (CO_2_) ↑↓ Primary productivity/respiration	↓pH	Affecting aquatic organisms adapted to a specific pH range. ↓Shellfish production	[51,58,62,63]

## Data Availability

The datasets used and/or analysed during the current study are available from the corresponding author on reasonable request.
